# Internal mammary artery dilatation in a patient with aortic coarctation, aortic stenosis, and coronary disease. Case report

**DOI:** 10.1186/1749-8090-6-55

**Published:** 2011-04-17

**Authors:** Jose Rubio Alvarez, Laura Reija Lopez, Juan Sierra Quiroga, Jose M Martinez Comendador, Anxo Martinez-de-Alegria, Jose M Martinez Cereijo, Cristian Delgado Dominguez

**Affiliations:** 1 Department of Cardiac Surgery, Universitary Hospital Santiago de Compostela (CHUS), SERGAS Travesia Choupana SN Santiago de Compostela, 15706 La Coruña, Spain; 2 Department of Radiology, Universitary Hospital Santiago de Compostela (CHUS), SERGAS Travesia Choupana SN Santiago de Compostela, 15706 La Coruña, Spain

## Abstract

The ideal surgical approach is unclear in adult patients with coarctation of the aorta that is associated with other cardiovascular pathologies that require intervention. Standard median sternotomy allows simultaneous, coronary revascularization surgery, valve replacement and repair of aortic coarctation. However the collateral circulation and the anatomy of the mammary arteries must be determined, to avoid possible complications. We report a case of a 69 year-old man with aortic coarctation, aortic stenosis, coronary artery disease and internal mammary artery dilatation who underwent concomitant surgical procedures through a median sternotomy.

## Background

The ideal surgical approach is unclear for adults patients with aortic coarctation whom have other associated cardiovascular pathologies. These patients have extensive collateral circulation, mainly from branches of the subclavian artery [1]. Long-term complications include aneurysmal dilatation of collateral circulation, which may eventually rupture. This can be a problem when a median sternotomy is performed.

## Case Report

**Figure 1:**
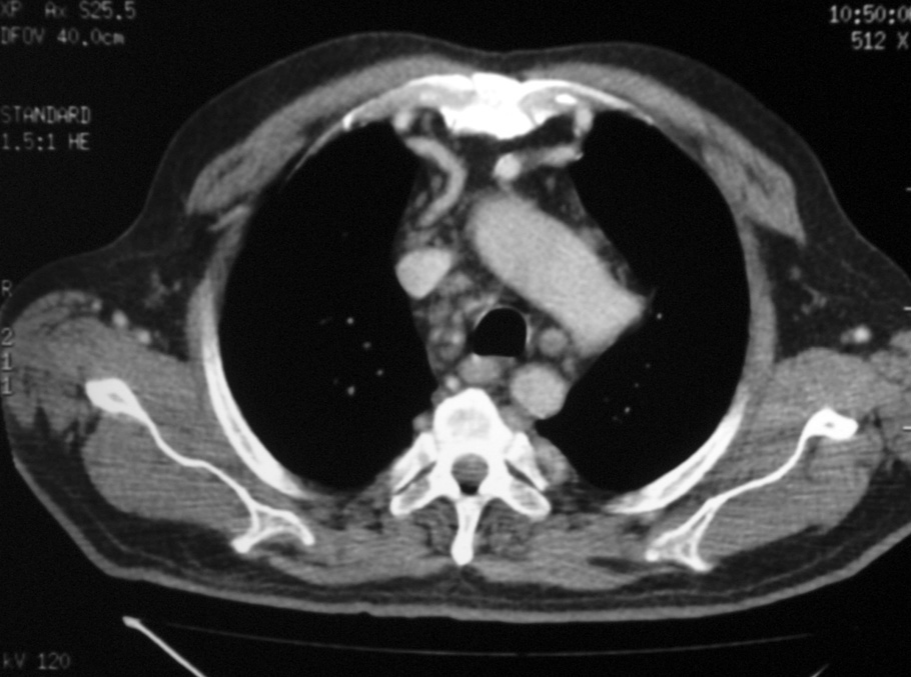
**Computerized tomography showing bilateral internal mammary artery dilatation**.

A 69 year-old man was admitted to our hospital complaining of congestive heart failure. His medical history included long-standing hypertension. On admission the patient had a blood pressure of 180/70 mmHg with diminished femoral pulses. Electrocardiographic analysis showed left ventricular hypertrophy and left bundle branch block. Transthoracic echocardiography showed an 80 mm Hg peak gradient and 0,8 cm2 surface area in the aortic valve. The left ventricular ejection fraction was 35%. Coronary angiography revealed severe disease of the left anterior descending coronary artery, and the coarctation was confirmed by aortography. There was a severe coarctation just distal to the left subclavian artery. Computerized tomography angiography to determine the anatomy and severity of the coarctation, showed bilateral internal mammary artery dilatation (Figure 1).

**Figure 2:**
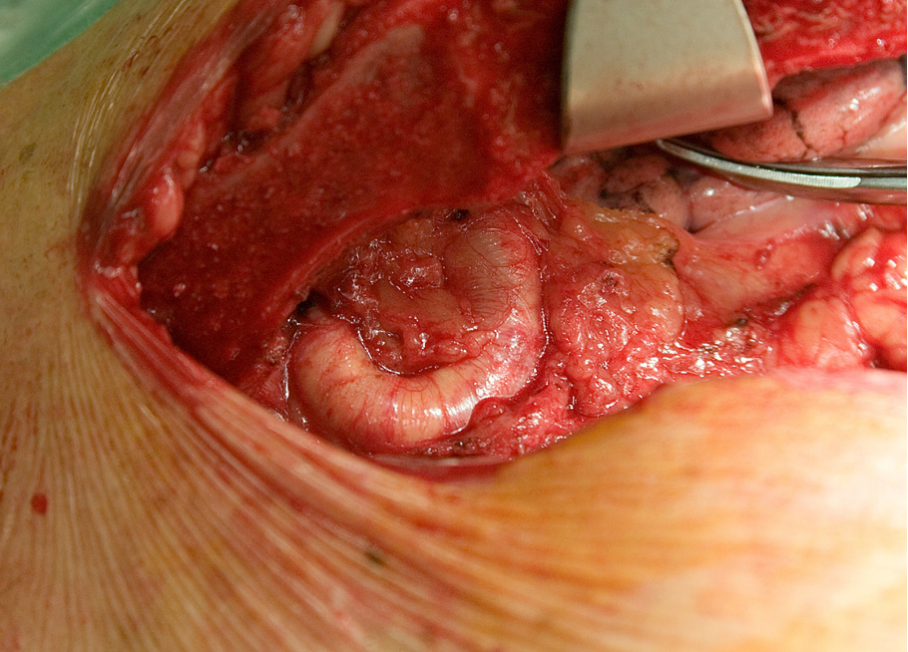
**Aneurysmal dilatation of the left internal mammary artery close to the sternum**.

Standard median sternotomy was performed using a vibrating saw. A large internal mammary artery was observed that was unsuitable for use as conduit for revascularization (Figure 2). Cardiopulmonary bypass was performed using high arterial cannulation in the ascending aorta and single venous cannulation in the right atrial. We used systemic cooling to a temperature of 33° and antegrade cardioplegia was administered for myocardial protection. Before cardioplegic arrest, the heart was retracted and the posterior pericardium was exposed, the descending thoracic aorta and the esophagus were palpated and the pericardium was opened. Following cardioplegic arrest the coronary artery bypass grafting was performed with a saphenous vein to the left anterior descending coronary artery. After revascularization, the heart was retracted and a partial occlusion clamp was applied to the anterior wall of the descending thoracic aorta where an end-to-side anastomosis with a 22 mm vascular graft was constructed using 3-0 polypropylene running suture. The graft was routed anterior to the esophagus, posterior to the inferior vena cava, and anterior to the right inferior pulmonary vein. After performing the distal anastomosis of the vascular graft to the descending aorta, an aortic valve replacement was performed using a 23 mm Sorin Bicarbon prosthesis. The native bicuspid aortic valve was very calcified. The aortic clamp was removed, the vascular graft was cut obliquely and a proximal anastomosis was fashioned end-to-side to the external side of the ascending aorta using a partial occlusion clamp. The proximal anastomosis of the saphenous vein was performed over a different partial occluding clamp. The patient came off bypass with minimal vasoconstrictor support. Cardiopulmonary bypass and myocardial ischaemic time were 135 and 80 minutes respectively. The postoperative period was uneventful, and the patient was discharged on his tenth postoperative day on hydrochlorothiazide, enalapril and Dicumarine. Three years later, he is classified as having NYHA class I.

**Figure 3:**
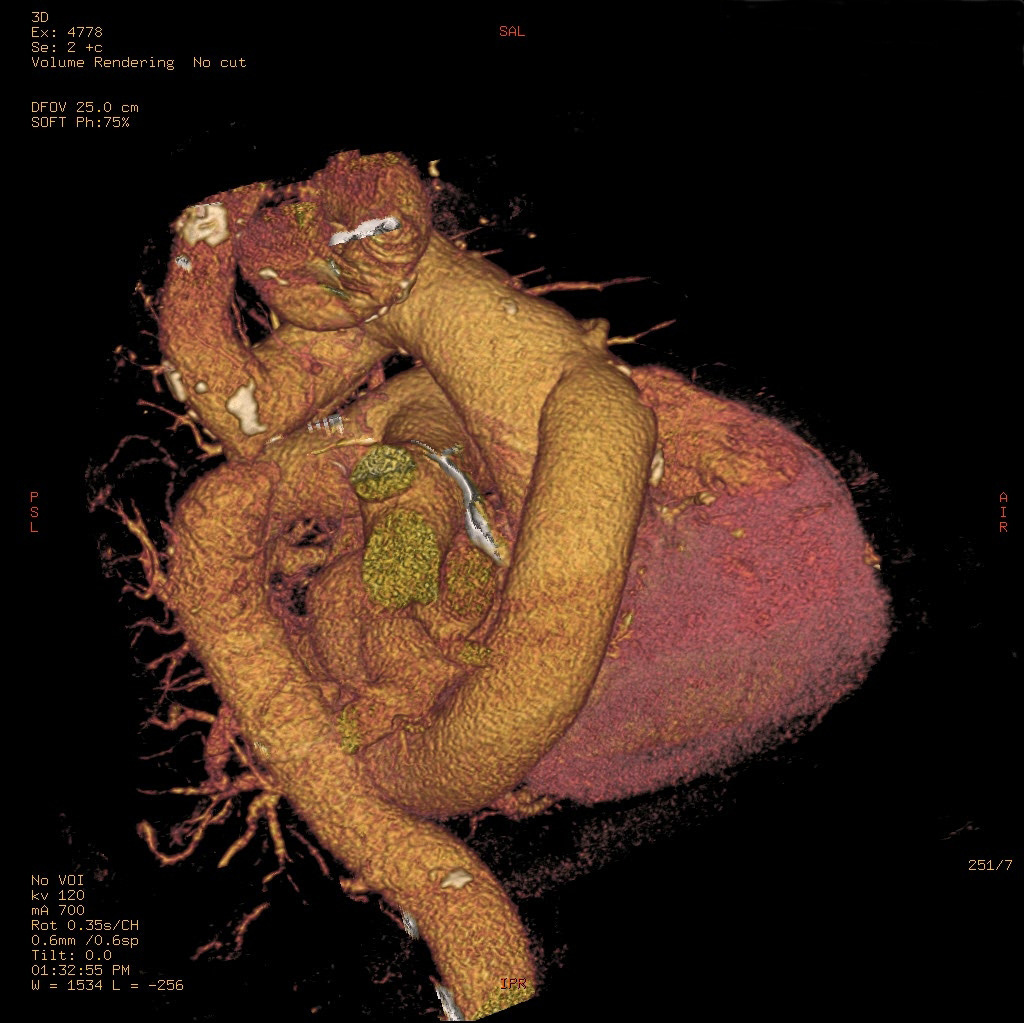
**Computerized tomography with volume rendering reconstruction showing the vascular graft between the ascending aorta and descending thoracic aorta**. The aortic coarctation is visualized.

**Figure 4:**
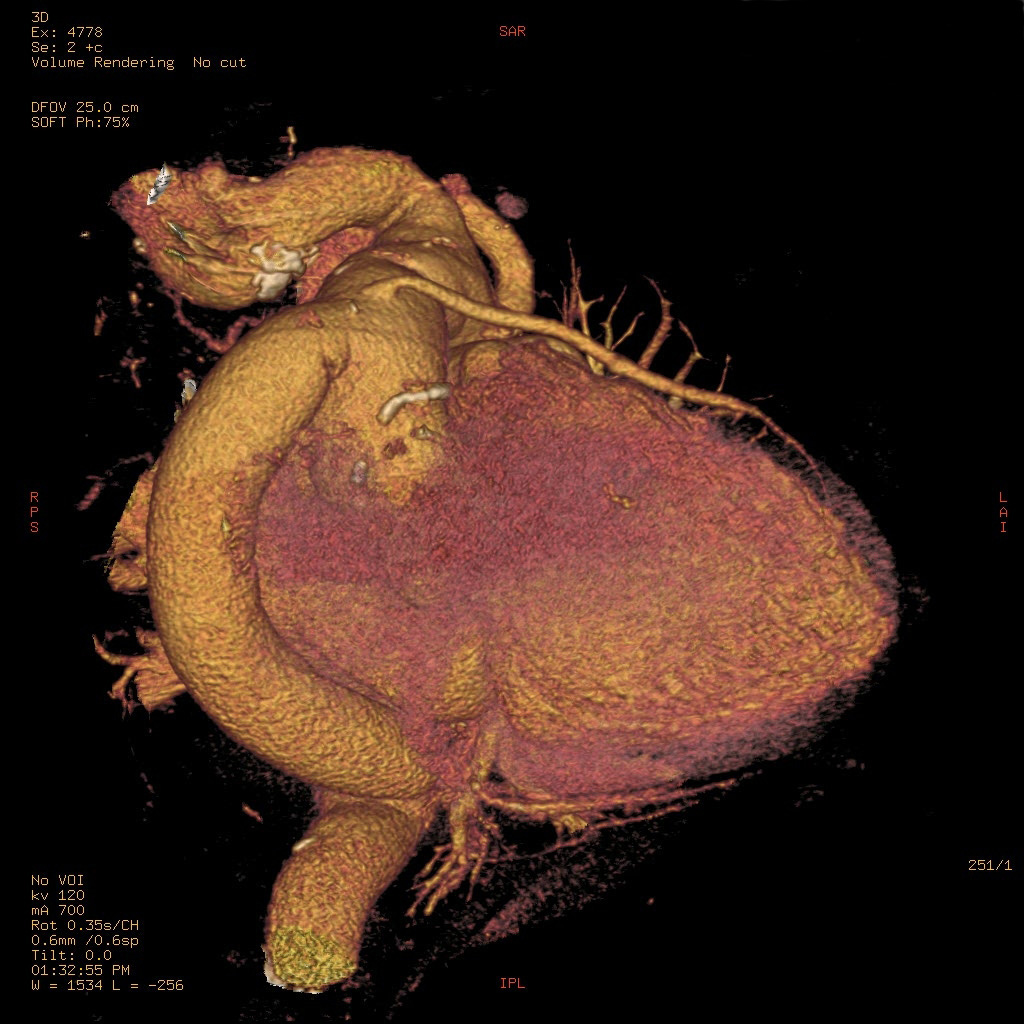
**Computerized tomography with volume rendering reconstruction showing the coronary artery bypass graft with a saphenous vein to the left anterior descending coronary artery**.

A volume rendering reconstruction of the postoperative computerized tomography scan showed a vascular graft between the ascending aorta and descending thoracic aorta and the aortic coarctation (Figure 3) and the bypass to the left anterior descending coronary artery (Figure 4).

## Discussion

Although coarctation of the aorta is a congenital heart defect, it is frequently diagnosed in adulthood because patients can remain symptom-free for many years. However, hypertension is common and more severe in older than in younger patients, and as in this case study, older patients with coarctation of the aorta may present with heart failure. Collateral circulation between the part of the aorta proximal to the coarctation and that distal to it, it is one of the striking features of coarctation. There is usually extensive collateral circulation in adults with coarctation, and the source is mainly due to branches of both subclavian arteries, particularly the internal thoracic and vertebral arteries. In these patients, long-term complications include aortic aneurysm formation and aneurysmal dilatation of the collateral circulation, which may eventually rupture [1]. Overall the prevalence of aneurysm is about 10% by the end of the second decade of life, 20% by the end of the third decade, and probably even higher in older patients [2]. When an adult patient with aortic coarctation needs median sternotomy for cardiac surgery, Laks et al [1] suggested that magnetic resonance angiography with three dimensional computerized reconstruction to assess the transverse arch, isthmus, and descending aorta is often useful. We think it is necessary to define the anatomy and severity of the coarctation as well as the size of mammary arteries, because these may be damaged by the saw. In the patient presented here, we decided to use the vibrating saw because the computerized tomography showed a very large mammary artery near the sternum.

Approximately 25 to 50 percent of patients with coarctation have bicuspid aortic valves, and these valves have a tendency to calcify in adult life, producing aortic stenosis [3]. All of these lesions impose increased afterload on the left ventricle and, if severe and untreated, result in hypertrophy and failure of the left ventricle [4]. This was the case for our patient upon admission to the hospital.

In adult patients with aortic coarctation in combination with other cardiac diseases, surgical management may be complicated, and there is no consensus on the optimal approach. The long-term outcome is known only after surgery and although stent therapy has been proven efficient, relatively simple and free adverse events in the majority of cases, the equivalence of the endovascular repair in the long-term has not yet been determined [5]. Some authors have suggested a single-stage repair [6-8], while exposure of the descending aorta through a median sternotomy and the posterior pericardium was described by Vijayanagar et al [9] in a patient with aortic coarctation associated with aortic valve regurgitation. In that patient, the vascular graft was placed around the left margin of the heart and was anastomosed proximally to the anterior wall of the ascending aorta. Powell et al [10] described a modification of this technique in which the graft is routed around the right margin of the heart and anastomosed proximally to the right lateral ascending aorta. Routing the graft behind the inferior vena cava but anterior to the right inferior pulmonary vein may protect the graft if reoperation is necessary.

## Conclusions

We conclude that adult patients with aortic coarctation combined with other cardiac pathologies may be treated with a single-stage repair. Because median sternotomy is the best approach, we believe that to minimize morbidity, a computerized tomography angiogram should be performed prior to surgery to identify possible aneurysmal dilatation of vessels involved in collateral circulation.

## Consent

Written informed consent was obtained from the patient for publication of this case report and accompanying images. A copy of the written consent is available for review by the Editor-in-Chief of this journal.

## Competing interests

The authors declare that they have no competing interests.

## Authors' contributions

LRL drafted the manuscript. JRA and JSQ conceived the study and performed the operation. AMDA performed the radiologic study. JMMC provided patient follow-up data. JMMC participated in the manuscript preparation. CDD Collected references. All authors read and approved the final manuscript.
